# The relationship between perception of the functional training environment and athletes’ training engagement: the mediating role of psychological safety

**DOI:** 10.3389/fpsyg.2026.1833341

**Published:** 2026-07-08

**Authors:** Hua Li

**Affiliations:** Department of Physical Education, Fujian Business University, Fuzhou, China

**Keywords:** environmental awareness, functional training scenarios, psychological safety, risk perception, training engagement

## Abstract

**Introduction:**

Functional training is often characterized by uncertainty, exploratory movement, repeated trial and error, and the possibility of failure. In such contexts, understanding how athletes remain engaged despite these challenges is important for explaining training behavior. Drawing on Self-Determination Theory, Psychological Safety Theory, and Work Engagement Theory, this study examined the relationships among perceived functional training environment, psychological safety, and training engagement.

**Methods:**

Cross-sectional questionnaire data were collected from 468 active athletes, including members of high-level university sports teams and provincial professional training teams. Participants were drawn from multiple sports, including basketball, soccer, track and field, badminton, table tennis, and taekwondo. Structural equation modeling was used to test the proposed mediation model, and multigroup analysis was conducted to examine whether the model differed across gender groups.

**Results:**

Perceived functional training environment was positively associated with both psychological safety and training engagement. Psychological safety showed a significant indirect association between perceived functional training environment and training engagement. Multigroup analysis indicated that the overall model structure was stable across gender groups, although the strength of specific pathways differed. Female athletes showed stronger path coefficients for the pathways from perceived functional training environment to psychological safety and from psychological safety to training engagement.

**Discussion:**

These findings suggest that, in high-uncertainty functional training contexts, athletes’ psychological interpretation of risk, trial and error, and possible failure may help explain how training environments are associated with sustained training engagement. The study provides empirical evidence for clarifying the psychological pathway through which perceived functional training environments may support athletes’ engagement in training.

## Introduction

Training philosophy in competitive sport has increasingly shifted from isolated physical conditioning toward movement integration and situational adaptation. Within this shift, the role of training environments in shaping athletes’ psychological experiences and behavioral engagement has received growing scholarly attention (Bompa and Buzzichelli, 2019). Functional training, which emphasizes multi-joint coordination, task-oriented practice, and kinetic-chain integration, has been widely adopted to enhance body control, movement coordination, and adaptive capacity (Boyle, 2016; Cook, 2010). Compared with traditional isolated muscle training, functional training is characterized by greater task complexity, uncertainty, and exploratory movement demands. Athletes are therefore required not only to complete prescribed tasks but also to refine their movement strategies through repeated trial and error, feedback, and adjustment. These characteristics may be related to athletes’ psychological appraisal of the training context and their engagement in training.

Research in exercise psychology has shown that athletes’ subjective perceptions of the training context are closely associated with their level of training engagement (Appleton et al., 2016; Isoard-Gautheur et al., 2012). Training environments characterized by clear task demands, appropriately challenging activities, and supportive feedback have been associated with positive training experiences and sustained engagement (Guo et al., 2022). Self-Determination Theory further suggests that the satisfaction of athletes’ needs for autonomy, competence, and relatedness is central to the quality of motivation and training participation (Ryan and Deci, 2017; Van Yperen et al., 2025). Existing studies, however, have mainly examined general supportive climates, coaching behaviors, and team relationships. Less attention has been paid to the psychological mechanisms through which athletes respond to functional training environments marked by uncertainty, trial-and-error learning, and potential performance exposure.

Psychological Safety Theory provides a useful framework for addressing this issue. Psychological safety refers to an individual’s perception that they can express opinions, attempt new behaviors, and take interpersonal or task-related risks without fear of negative evaluation or punishment (Edmondson, 1999). This construct has gained increasing relevance in sport psychology, as athletes’ perceptions of supportive climates, team relationships, and coaching behaviors may influence their willingness to communicate, engage in learning, and regulate emotional experiences (Fransen et al., 2020; Taylor et al., 2022). In functional training contexts, psychological safety is closely linked to athletes’ perceived freedom to experiment, make errors, and expose movement limitations during practice. It may therefore serve as a crucial psychological mechanism linking perceived training environment to training engagement.

Training engagement is commonly conceptualized as a multidimensional psychological state involving vigor, dedication, and absorption (Schaufeli et al., 2002). Previous studies have shown that engagement in sport and training settings is shaped by environmental characteristics, motivational climate, coaching behaviors, and the satisfaction of basic psychological needs (Liu et al., 2024; Luo et al., 2025). Nevertheless, most available evidence has been derived from general training or team contexts. For exploratory and uncertain tasks such as functional training, systematic empirical evidence remains limited regarding how athletes’ perceptions of the training environment are associated with psychological safety and, in turn, training engagement.

Accordingly, this study introduces the construct of perceived functional training environment, defined as athletes’ overall evaluation of task clarity, challenge appropriateness, and feedback support in functional training. Psychological safety is examined as a key mediating mechanism linking perceived functional training environment to training engagement. The study also investigates whether these pathways differ by gender. By doing so, this research extends the explanatory framework of athletes’ training engagement to the task-context level and provides empirical evidence for the role of psychological safety in high-uncertainty training conditions.

## Theoretical foundations and research hypotheses

### Theoretical foundations

#### The generative logic of athletes’ autonomy experience in task-challenge contexts (self-determination theory)

Self-Determination Theory, developed by Deci and Ryan in the 1980s, proposes that the persistence and vitality of human behavior are shaped by the quality of motivation. Intrinsic motivation, in particular, is closely associated with the satisfaction of three basic psychological needs: autonomy, competence, and relatedness (Deci and Ryan, 2000). When individuals perceive autonomy in action, receive competence-affirming feedback, and experience positive interpersonal connection within a given activity context, they are more likely to develop intrinsic motivation and sustain behavioral engagement (Ryan and Deci, 2017). Self-Determination Theory therefore provides a useful framework for explaining why individuals differ in their motivation and participation across specific contexts.

In competitive sport training, the structure of training tasks and the organization of the training environment are key contextual factors that shape athletes’ motivational experiences. Compared with traditional approaches that rely primarily on repetitive movement drills, functional training integrates multi-joint actions, situational tasks, and movement-control demands, thereby increasing task complexity and exploratory requirements (Boyle, 2016). In such contexts, athletes are required not only to complete prescribed training tasks but also to develop adaptive movement solutions while continuously adjusting bodily control and movement strategies. This combination of task orientation and exploratory practice may provide athletes with greater behavioral autonomy and more opportunities to demonstrate competence, thereby strengthening their perceived autonomy and competence during training.

Empirical evidence supports the role of training contexts in shaping athletes’ motivational experiences. Studies have shown that athletes who perceive higher levels of autonomy support and task challenge within the training environment are more likely to develop intrinsic motivation, training engagement, and sustained participation (Ntoumanis, 2012). In addition, the motivational climate created by coaches and the structure of training tasks may influence athletes’ training behavior by shaping the extent to which their basic psychological needs are satisfied (Appleton et al., 2016). In exploratory and challenging training contexts, athletes may experience competence development and task meaningfulness, which can further enhance their internal identification with training and their willingness to remain engaged.

Taken together, Self-Determination Theory offers a central explanatory framework for understanding how training contexts shape athletes’ behavior. From this perspective, the training environment is not merely a physical setting for developing fitness and technical skills, but also a contextual system that structures athletes’ psychological experiences. By creating task conditions that involve challenge, exploration, and adaptive problem solving, functional training may support athletes’ sense of autonomy and competence, thereby helping to explain the motivational basis of training participation. However, in the present study, Self-Determination Theory is used primarily to explain the theoretical link between training contexts and athletes’ positive psychological experiences. Autonomy, competence, and relatedness need satisfaction were not directly measured. Accordingly, basic psychological need satisfaction is not specified as an empirical mediating variable in the proposed model; rather, it serves as a theoretical foundation for interpreting the potential psychological significance of the perceived functional training environment.

#### The contextual mechanism of psychological safety formation in team interaction climate (psychological safety theory)

The concept of psychological safety was first introduced by Kahn (1990) in organizational behavior to describe a psychological state in which individuals feel able to express themselves and take interpersonal risks without fear of negative evaluation or punishment. Edmondson (1999) subsequently extended this concept in research on team learning, defining psychological safety as team members’ shared perception that the interactive environment is safe for expressing views, asking questions, admitting mistakes, and attempting new behaviors. Accordingly, psychological safety does not merely denote a generally positive atmosphere. Rather, it refers to the sense of security that individuals develop when facing uncertainty, risk exposure, and evaluative pressure.

In competitive sport training, athletes’ behaviors occur within contexts that are both highly interactive and strongly evaluative. Athletes are required to attempt new movement patterns, refine technical details, and respond to feedback from coaches and teammates. These processes often involve the possibility of failure, the exposure of ability limitations, and external evaluation. This is particularly evident in functional training, where tasks usually involve considerable movement complexity and exploratory demands. Athletes must improve performance through repeated trial and error, movement correction, and adaptive adjustment. When the training environment lacks inclusiveness, or when mistakes are interpreted as evidence of insufficient ability, athletes may become less willing to express themselves, attempt unfamiliar tasks, or remain fully engaged. Conversely, training contexts that tolerate errors, encourage exploration, and provide constructive feedback are more likely to foster psychological safety, thereby supporting an open learning orientation and sustained training participation.

Existing research suggests that psychological safety facilitates learning behavior, active expression, and sustained participation in team settings (Edmondson and Lei, 2014). In sport contexts, athletes’ perceptions of supportive climates, team relationships, and coaching behaviors may also influence their willingness to communicate, engage in learning, and regulate emotional experiences (Fransen et al., 2020). These findings indicate that, in competitive sport environments characterized by high performance demands and frequent evaluation, psychological safety is associated not only with interpersonal relationship quality but also with athletes’ willingness to attempt new tasks, disclose limitations, and correct errors during training. Psychological safety is especially important in functional training because this form of training inherently involves uncertainty, movement exploration, and the possibility of failure. Athletes are more likely to allocate psychological resources to the task itself when they perceive that mistakes are tolerated, experimentation is permitted, and feedback is intended to support improvement rather than criticism.

It is also necessary to distinguish psychological safety from the basic psychological needs proposed in Self-Determination Theory. Although psychological safety is related to autonomy, competence, relatedness, and broader contextual support, these constructs are not conceptually identical. Autonomy mainly concerns whether athletes perceive choice and volitional participation in training. Competence refers to athletes’ sense of mastery, progress, and effective control. Relatedness emphasizes feelings of acceptance, understanding, and positive connection with others. Psychological safety, by contrast, focuses on whether athletes feel safe to express views, reveal limitations, attempt new movements, and acknowledge mistakes during training interactions without fear of humiliation, exclusion, punishment, or negative evaluation. In functional training contexts, autonomy, competence, and relatedness help explain why athletes are willing to participate, whereas psychological safety further explains why they dare to persist in tasks marked by uncertainty, trial-and-error learning, and evaluative pressure.

In summary, Psychological Safety Theory provides an important explanatory framework for understanding athletes’ psychological experiences in functional training contexts. Athletes’ psychological safety during training arises not only from team interaction, coach feedback, and supportive climates but also from their subjective judgments about errors, risks, and the consequences of failure. Treating psychological safety as a distinct psychological variable, rather than as a direct equivalent of general motivational need satisfaction, helps clarify how perceptions of the functional training environment may influence training engagement through risk buffering and safety appraisal.

#### The behavioral pathways linking positive psychological experience to sustained training engagement (work engagement theory)

Work engagement was first proposed by Schaufeli et al. (2002) in the field of organizational behavior to describe a positive psychological state experienced during work-related activities. Its core dimensions include vigor, dedication, and absorption. Specifically, vigor refers to high levels of energy and sustained effort during task performance; dedication reflects a strong sense of meaning, enthusiasm, and identification with the task; and absorption denotes a state of deep concentration and immersion during task execution.

In competitive sport training, athletes’ training behavior can be understood as a continuous process of task engagement. Training engagement refers to athletes’ energetic investment, attentional focus, and psychological identification with training tasks. Similar to engagement in general work contexts, athletes who experience positive psychological states during training are more likely to sustain effort and maintain high levels of behavioral participation. Thus, training engagement reflects not only athletes’ observable involvement in training but also their motivational orientation and psychological identification with the training context.

Relevant research indicates that positive psychological experiences provide an important foundation for individual engagement. Bakker and Demerouti (2008) argued that individuals are more likely to demonstrate positive work engagement when they perceive support, trust, and psychological safety within a task context. In sport research, athletes’ positive perceptions of the training environment have also been found to be associated with training participation and sustained effort (Lonsdale et al., 2007). Athletes who experience psychological safety and contextual support during training may be more likely to maintain concentration, persist in skill learning, and invest effort in task execution, which may be associated with stronger behavioral engagement.

In summary, Work Engagement Theory provides a useful framework for understanding sustained participation in task-based contexts. From this perspective, training engagement can be regarded as a positive psychological and behavioral state that emerges within the training environment. The formation of this state depends, in part, on athletes’ psychological experiences during training. When athletes perceive a high level of psychological safety, they are more likely to maintain a positive orientation toward training and remain consistently engaged in training tasks. Therefore, incorporating psychological safety as a key psychological mechanism is theoretically justified when examining the relationship between the perceived functional training environment and athletes’ training engagement.

### Research hypotheses

Based on the theoretical analysis above, this study defines perceived functional training environment as athletes’ overall evaluation, formed during functional training tasks, of task clarity, challenge appropriateness, and the extent to which feedback supports movement correction and sustained effort. This construct reflects athletes’ situational appraisal within highly integrated and adaptive training tasks. Functional training requires substantial motor exploration and involves a high likelihood of error exposure. Whether athletes remain consistently engaged in such training may depend largely on how they interpret the structural features of training tasks and their psychological implications. Accordingly, this study proposes a set of hypotheses concerning the relationships among perceived functional training environment, psychological safety, and training engagement, while further examining whether these relationships differ across gender groups.

#### H1. Perceived functional training environment and psychological safety

According to Self-Determination Theory, task structure and contextual support within the training environment shape individuals’ psychological experiences. When a training context provides opportunities for exploration and competence-related feedback, individuals are more likely to develop positive psychological perceptions (Deci and Ryan, 2000). In competitive sport training, athletes’ experiences in functional training may be related to task-oriented practice and movement challenges. Therefore, athletes’ positive perceptions of the functional training environment may be associated with higher psychological safety.

*H1a*. A more positive perceived functional training environment is associated with higher psychological safety among athletes.

*H1b*. Gender may moderate the relationship between perceived functional training environment and psychological safety.

#### H2. Psychological safety and training engagement

Work Engagement Theory suggests that individuals are more likely to demonstrate higher levels of behavioral engagement when they develop positive psychological experiences within a task context (Schaufeli et al., 2002). In competitive sport training, psychological safety may be associated with lower psychological pressure during trial-and-error learning and with greater training participation and sustained effort.

*H2a*. Higher psychological safety is associated with higher training engagement among athletes.

*H2b*. Gender may moderate the relationship between psychological safety and training engagement.

#### H3. Perceived functional training environment and training engagement

Research on training environments indicates that individuals’ subjective appraisals of activity contexts are closely related to their participation behavior. When athletes perceive the training environment as challenging, meaningful, and supportive, they are more likely to sustain effort and remain engaged in training (Ntoumanis, 2012).

*H3a*. A more positive perceived functional training environment is associated with higher training engagement among athletes.

*H3b*. Gender may moderate the relationship between perceived functional training environment and training engagement.

#### H4. The mediating role of psychological safety

Integrating Self-Determination Theory and Psychological Safety Theory suggests that the training environment may be linked to athletes’ behavior both directly and indirectly through psychological experiences. When athletes develop a high level of psychological safety during training, they are more likely to maintain a positive state of engagement and persist in training tasks.

*H4a*. Psychological safety mediates the relationship between perceived functional training environment and training engagement.

**Figure 1:**
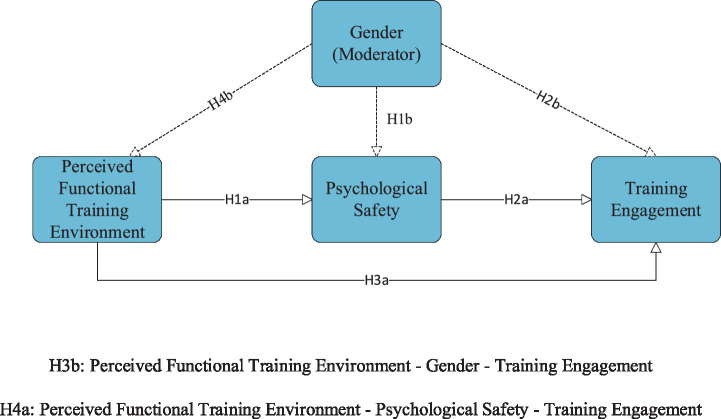
Conceptual model of the study.

In summary, the hypothesized model constructed in this study is presented in Figure 1.

## Research design

### Study population and sample sources

This study employed stratified cluster sampling. The target population consisted of athletes currently training within China’s university and provincial competitive sport training systems. Sample selection focused on competitive training settings with stable training routines and clearly defined training objectives. This design enabled the study to examine athletes’ subjective perceptions of the functional training environment, together with their psychological and behavioral responses, within relatively standardized and high-intensity training contexts. The sample covered major regions, including East China, Central China, and South China, and included five provincial-level administrative regions: Jiangsu, Zhejiang, Hubei, Hunan, and Guangdong. Participants were mainly recruited from high-level university sports teams and provincial professional training teams. These training systems share similar organizational structures, training-load arrangements, and coaching management models, which facilitated the examination of the proposed theoretical model within a relatively unified institutional context and helped reduce the potential influence of contextual heterogeneity on the relationships among variables.

The formal survey was administered through a combination of in-person distribution and online questionnaires. A total of 520 questionnaires were distributed, and 468 valid responses were obtained, yielding a valid response rate of 90.0%. The preliminary test sample, which included 120 athletes, was independent of the formal survey sample and was not included in the subsequent structural equation modeling analysis. During data screening, invalid questionnaires were excluded if they showed excessively short completion times, repetitive or patterned responses, or missing values for key variables. Before data collection, the research protocol was reviewed and approved by the Academic Ethics Committee of Fujian Business University (No. SYLL20250101). Adult participants provided written or electronic informed consent. For participants under the age of 18, written informed consent was waived by the ethics committee; oral informed consent was obtained from their legal guardians, and assent was obtained from the participants themselves before questionnaire completion. All participants completed the applicable consent or assent procedure after being fully informed of the study objectives, anonymity and confidentiality procedures, voluntary participation, and their right to withdraw at any time. The entire research process followed academic ethical standards to ensure appropriate data collection, storage, and use.

Several procedural controls were implemented to reduce the potential influence of common method bias. Before completing the questionnaire, participants were informed that the study was conducted solely for academic purposes, that all responses would remain anonymous, that there were no right or wrong answers, and that their responses would not affect their individual training evaluations. These procedures were intended to reduce social desirability bias and evaluation concerns. In addition, items measuring different constructs were dispersed throughout the questionnaire, and clear, accessible wording was used to minimize response consistency caused by item misunderstanding. During data organization, questionnaires with excessively short completion times, patterned responses, or missing key variables were further removed to improve data quality and reduce the risk of common method bias.

In the valid sample, athletes ranged in age from 17 to 25 years (*M* = 20.3, SD = 1.9). The sample included 256 male athletes (54.7%) and 212 female athletes (45.3%). Training experience ranged from 3 to 12 years (*M* = 7.1, SD = 2.6). The sample covered basketball, soccer, track and field, badminton, table tennis, taekwondo, and other sports. In terms of sport category, endurance sports accounted for approximately 34.2%, skill-based sports for 39.5%, and strength and contact sports for 26.3%. Regarding athletic ranking, 102 participants were National Class I athletes (21.8%), 198 were National Class II athletes (42.3%), and the remaining participants were either National Class III athletes or unranked athletes (35.9%). Overall, the sample showed adequate diversity in gender composition, sport distribution, and training experience, providing a stable empirical basis for the subsequent structural equation modeling analysis.

Although stratified cluster sampling across multiple regions and teams was used to enhance sample coverage and training-context diversity, the official questionnaire did not fully record school, team, or specific training-group codes in order to protect participant anonymity. As a result, the subsequent analyses were conducted primarily at the individual level using structural equation modeling rather than multilevel modeling.

### Measurement instruments

Based on the variable structure of the theoretical model, this study developed or selected measurement instruments for three latent variables: perceived functional training environment, psychological safety, and training engagement. All scales used a 5-point Likert response format to ensure comparability across structural variables and suitability for subsequent structural equation modeling. Response options ranged from 1 = “strongly disagree” to 5 = “strongly agree.” Scores for each variable were calculated by averaging the corresponding items, with higher scores indicating higher levels of the measured construct.

Perceived functional training environment was used to assess athletes’ subjective appraisal of the organizational characteristics of functional training tasks. Given that functional training emphasizes movement integration, continuous adjustment, and trial-and-error correction, this construct was defined as athletes’ overall evaluation of task comprehensibility, challenge manageability, and the corrective value of feedback. It was operationalized into three dimensions: task clarity, challenge appropriateness, and feedback support. The construct was therefore limited to athletes’ perceptions of the organizational features of functional training tasks and did not include coach support, team relationships, or the general training atmosphere. Specifically, task clarity refers to athletes’ understanding of training objectives, movement requirements, and performance criteria. Challenge appropriateness reflects whether task complexity and difficulty remain within a range that promotes progress without causing an excessive loss of control. Feedback support refers to whether information received during training provides clear guidance for movement correction, error adjustment, and continued effort. Because no established scale was available for assessing this construct in competitive functional training contexts, this study developed the scale on the basis of functional training theory (Boyle, 2016; Cook, 2010) and relevant research on training environments.

The development of the scale involved construct definition, qualitative data synthesis, item generation, expert review, pilot testing, and validation with the formal sample. Following the literature review, the research team conducted semi-structured interviews to identify key experiences influencing athletes’ task comprehension, challenge acceptance, and movement correction in functional training. The interviewees included 10 active athletes with systematic functional training experience and four coaches with extensive experience in physical conditioning or sport-specific training. Athlete interviews focused on task comprehension, perceived movement difficulty, error exposure, feedback reception, and willingness to persist in functional training. Coach interviews focused on task design, movement goal setting, difficulty control, feedback methods, and athletes’ common psychological responses. Each interview lasted approximately 20–35 min and was recorded and transcribed with participants’ informed consent.

The interview data were analyzed using thematic analysis. The research team first conducted open coding of the interview transcripts to extract statements related to perceived functional training environment, such as whether training goals were clear, movement standards were explicit, task difficulty was manageable, adjustments were allowed during training, and feedback helped improve movement quality. Similar codes were then compared, merged, and organized into three themes: task objectives and movement requirements, training difficulty and ability matching, and feedback information and movement correction. In combination with functional training theory and research on training environments, these themes were further generalized into the dimensions of task clarity, challenge appropriateness, and feedback support. Based on the literature review and interview findings, an initial pool of 14 items was generated to capture athletes’ perceptions of task objectives, movement requirements, difficulty matching, error correction, and feedback support during functional training.

Three experts were subsequently invited to review the item pool on the basis of their professional expertise, research experience, and familiarity with competitive training practice. The expert review focused on three aspects. First, the experts assessed the consistency between item content and construct definitions, particularly whether the items reflected the organizational characteristics of functional training tasks. Second, they evaluated the clarity of item wording, with attention to semantic redundancy, ambiguity, and potential interpretive confusion. Third, they examined the contextual suitability of the items, especially whether the wording corresponded to functional training practices in competitive sport. Based on expert feedback, the initial 14 items were merged, reduced, and revised. Three items were deleted: one because it overlapped semantically with an item in the task clarity dimension, one because it resembled general coaching support rather than the characteristics of functional training tasks, and one because its situational focus was insufficiently specific and could be interpreted as an evaluation of the general training atmosphere. After expert review, 11 items were retained for pilot testing.

During the pilot testing phase, exploratory factor analysis was conducted with an independent sample of 120 active athletes. This pilot sample was not included in the formal survey or in the subsequent structural equation modeling analyses. The exploratory factor analysis showed that the overall structure of the scale was generally consistent with the three predefined dimensions. However, two items failed to meet the retention criteria. One item had a factor loading below 0.50, and the other showed high cross-loadings on two dimensions, with a loading difference of less than 0.20, indicating unclear dimensional affiliation. Considering factor loadings, cross-loadings, item representativeness, and dimensional balance, these two items were removed. The final Functional Training Environment Perception Scale consisted of nine items across three dimensions: task clarity, challenge appropriateness, and feedback support. The pilot results showed that the three dimensions cumulatively explained 67.84% of the total variance, and item factor loadings ranged from 0.64 to 0.83, indicating a clear preliminary factor structure.

Confirmatory factor analysis was then conducted using the formal sample to examine the three-dimensional structure of the scale. The results indicated that the three-factor model showed good fit: *χ*^2^/df = 2.41, CFI = 0.958, TLI = 0.946, RMSEA = 0.055, and SRMR = 0.041. Standardized factor loadings ranged from 0.63 to 0.81 and were all statistically significant (*p* < 0.001). The scale also demonstrated satisfactory internal consistency and convergent validity, with Cronbach’s *α* = 0.88, CR = 0.90, and AVE = 0.52. To further evaluate the construct structure, the three-factor model was compared with a one-factor model and several two-factor alternative models. The three-factor model showed superior fit, suggesting that task clarity, challenge appropriateness, and feedback support jointly contribute to perceived functional training environment while retaining distinct structural meanings. Together with the Fornell–Larcker criterion and HTMT results, these findings support the construct validity, convergent validity, and discriminant validity of the scale in the present sample. It should be noted, however, that the Functional Training Environment Perception Scale was developed specifically for the context of this study. Although the scale showed acceptable reliability and validity in the current sample, the path analysis results should be interpreted as preliminary evidence based on the present measurement framework and should be further validated in independent samples and different training systems. The final nine items are listed in the Appendix.

Psychological safety was measured using a revised version of Edmondson’s (1999) Team Psychological Safety Scale. Because the original scale was developed primarily for organizational and team management contexts, this study adapted the items according to the principle of preserving the original conceptual meaning while aligning the wording with competitive training contexts. Terms referring to general work teams were replaced with expressions relevant to sport training. For example, phrases such as “team members” and “work context” were adapted to “during training” and “in front of the coach and teammates” to better reflect athletes’ psychological experiences when attempting movements, expressing ideas, revealing weaknesses, and correcting errors. The revised scale retained seven items and used a 5-point Likert response format. Higher scores indicated stronger psychological safety during training. The complete items are provided in the Appendix. The measurement results showed good internal consistency and construct validity, with Cronbach’s *α* = 0.86, CR = 0.90, and AVE = 0.55.

Training engagement was measured using an adapted version of the Athlete Engagement Questionnaire developed by Lonsdale et al. (2007). Items were screened and revised to align with the present study’s focus on daily training contexts. During adaptation, the conceptual content of the three core dimensions—vigor, dedication, and focus—was retained. However, wording that was more closely related to general sport participation or competition states was revised to describe behaviors and psychological experiences in daily training. The final scale consisted of 12 items across three dimensions and used a 5-point Likert response format. Higher scores indicated higher levels of training engagement. The complete items are provided in the Appendix. Reliability and validity analyses indicated good measurement quality, with Cronbach’s *α* = 0.92, CR = 0.93, and AVE = 0.53.

To ensure the scientific rigor and semantic appropriateness of the measurement instruments, a small number of items with unclear wording were further revised based on the pilot test results before formal administration. Overall, Cronbach’s α values for all scales exceeded 0.80, CR values were greater than 0.85, and AVE values exceeded 0.50, indicating satisfactory internal consistency and convergent validity. These results suggest that the measurement instruments met the requirements for descriptive statistics, confirmatory factor analysis, and structural equation modeling. In addition, for scales derived from English-language literature, this study adopted a translation and back-translation procedure. First, two researchers with backgrounds in sport psychology independently translated the original items. A third bilingual researcher then conducted the back-translation, and the back-translated version was compared with the original scale to assess semantic consistency. Two field experts further evaluated the items for contextual appropriateness and conceptual equivalence, and specific wording was revised accordingly. This procedure helped ensure the applicability and semantic accuracy of the scales in competitive sport training contexts.

### Data analysis methods

Data analysis was conducted using SPSS 26.0 and AMOS 24.0. Before hypothesis testing, the sample data were screened and organized for preliminary analysis. Descriptive statistics were used to examine the distributional characteristics of each variable, and Pearson correlation analysis was performed to assess the direction and strength of the associations among perceived functional training environment, psychological safety, and training engagement. These analyses provided an empirical basis for subsequent model testing.

Structural equation modeling was then used to test the proposed theoretical model. Because the model included latent variables and their structural relationships, confirmatory factor analysis (CFA) was first conducted to evaluate the measurement model. This analysis examined the extent to which the observed indicators represented their corresponding latent constructs and assessed the adequacy of the overall measurement structure. For the newly developed Functional Training Environment Perception Scale, exploratory factor analysis was conducted using the pilot sample to examine its underlying dimensional structure. CFA was then performed using the formal sample to further validate the proposed three-dimensional measurement model. To evaluate construct validity, the three-factor model was also compared with a one-factor model and several two-factor alternative models. After the measurement model demonstrated acceptable fit, the structural model was estimated to examine the hypothesized paths among perceived functional training environment, psychological safety, and training engagement.

The mediating role of psychological safety in the relationship between perceived functional training environment and training engagement was examined using the bootstrap method. Indirect effects were tested with 5,000 bootstrap resamples, and 95% confidence intervals were calculated. An indirect effect was considered statistically significant when the confidence interval did not include zero.

Model fit was evaluated using multiple indices, including the chi-square-to-degrees-of-freedom ratio (*χ*^2^/df), Comparative Fit Index (CFI), Tucker–Lewis Index (TLI), Root Mean Square Error of Approximation (RMSEA), and Standardized Root Mean Square Residual (SRMR). Following the recommendations of Hu and Bentler (1999) and Kline (2016), χ^2^/df values below 3, CFI and TLI values above 0.90, preferably 0.95 or higher, RMSEA values below 0.06, with values up to 0.08 considered acceptable, and SRMR values below 0.08 were used as criteria for acceptable or good model fit.

Given the possibility that gender may influence the strength of the relationships among variables, multigroup structural equation modeling was conducted. Configural invariance and factor loading invariance were first tested across gender groups to determine whether the latent variable structure and primary measurement relationships were comparable. After basic measurement comparability was established, constraints were imposed on key structural paths, and constrained and unconstrained models were compared. Differences in model fit were evaluated using Δχ^2^, ΔCFI, and ΔRMSEA, following the criteria proposed by Cheung and Rensvold (2002). This procedure was used to examine whether the structural paths remained stable across gender groups.

## Research findings

### Descriptive statistics and correlation analysis

To preliminarily examine the distributional characteristics, measurement quality, and associations among the core variables, descriptive statistics, reliability analysis, convergent validity assessment, and Pearson correlation analysis were conducted for perceived functional training environment, psychological safety, and training engagement. The results are presented in Table 1.

**Table 1:** Descriptive statistics, reliability, validity, and correlations.

Variable	*M*	SD	Cronbach’s *α*	CR	AVE	1	2	3
ENV	3.34	0.89	0.88	0.90	0.52	–		
PS	3.46	0.94	0.86	0.90	0.55	0.58***	–	
ENG	3.49	0.93	0.92	0.93	0.53	0.63***	0.68***	—

ENV, functional training environment perception; PS, psychological safety; ENG, training engagement. CR, composite reliability; AVE, average variance extracted. ***p < 0.001.

As shown in Table 1, the mean scores for perceived functional training environment (*M* = 3.34, SD = 0.89), psychological safety (*M* = 3.46, SD = 0.94), and training engagement (*M* = 3.49, SD = 0.93) were all in the upper-moderate range. This suggests that athletes in the present sample generally reported relatively positive perceptions of the training environment, psychological safety, and training engagement. The Cronbach’s *α* values for all variables exceeded 0.85, composite reliability values were approximately 0.90, and average variance extracted values were above 0.50. These results indicate that the measurement instruments demonstrated satisfactory internal consistency and convergent validity.

Pearson correlation analysis showed that perceived functional training environment was significantly and positively associated with psychological safety (*r* = 0.58, *p* < 0.001) and training engagement (*r* = 0.63, *p* < 0.001). Psychological safety was also significantly and positively associated with training engagement (*r* = 0.68, *p* < 0.001). Overall, the directions of the correlations among the core variables were consistent with theoretical expectations, providing preliminary empirical support for the subsequent structural equation modeling analysis.

### Measurement model validation: confirmatory factor analysis (CFA)

To further examine the measurement structure of the three latent variables—perceived functional training environment, psychological safety, and training engagement—confirmatory factor analysis was conducted to test the proposed three-factor measurement model. The results showed that the theoretical model fit the data well: *χ*^2^/df = 2.37, CFI = 0.957, TLI = 0.949, RMSEA = 0.048, and SRMR = 0.042. These indices indicate that the measurement model demonstrated satisfactory fit to the sample data.

At the item level, all observed indicators loaded significantly on their corresponding latent variables (*p* < 0.001). Specifically, standardized factor loadings ranged from 0.63 to 0.81 for perceived functional training environment, from 0.66 to 0.82 for psychological safety, and from 0.64 to 0.84 for training engagement. These results suggest that the observed items adequately represented their respective latent constructs. The complete item-loading results are provided in the Appendix.

Given that the Functional Training Environment Perception Scale was newly developed for this study, additional model comparisons were conducted to assess the validity of its measurement structure. Specifically, the theoretical three-factor model was compared with a one-factor model and several two-factor alternative models. The model comparison results are presented in Table 2.

**Table 2:** Comparison of measurement models.

Model	*χ*^2^/df	CFI	TLI	RMSEA	SRMR
Three-factor model	2.37	0.957	0.949	0.048	0.042
One-factor model	8.17	0.694	0.651	0.123	0.109
Two-factor model A: ENV combined with PS	4.36	0.873	0.852	0.086	0.074
Two-factor model B: PS combined with ENG	4.71	0.858	0.834	0.091	0.079
Two-factor model C: ENV combined with ENG	4.28	0.881	0.861	0.083	0.071

ENV, functional training environment perception; PS, psychological safety; ENG, training engagement.

As shown in Table 2, the three-factor model demonstrated substantially better fit than the one-factor model and the three two-factor alternative models. The poor fit of the one-factor model indicates that the items could not be adequately explained by a single common construct. Although the two-factor models fit better than the one-factor model, their fit indices remained inferior to those of the theoretical three-factor model. These findings suggest that perceived functional training environment, psychological safety, and training engagement are empirically related but structurally distinct. The results therefore support the inclusion of these three constructs as separate latent variables in the structural equation model.

Discriminant validity was further examined using the Fornell–Larcker criterion and the heterotrait–monotrait ratio of correlations (HTMT). The results showed that the square root of the average variance extracted (AVE) for each latent variable was greater than its correlations with the other latent variables. In addition, all HTMT values were below 0.85, indicating acceptable discriminant validity among the constructs.

Because the data were collected using self-report questionnaires at a single time point, common method bias was also assessed. Harman’s single-factor test showed that unrotated exploratory factor analysis extracted five factors with eigenvalues greater than 1. The first factor explained 31.84% of the total variance, which was below the commonly used threshold of 40%. In addition, the one-factor CFA model showed poor fit: *χ*^2^/df = 8.17, CFI = 0.694, TLI = 0.651, RMSEA = 0.123, and SRMR = 0.109. This fit was markedly worse than that of the theoretical three-factor model.

Overall, the measurement model demonstrated satisfactory item loadings, model fit, construct distinctiveness, and control for common method bias. These results provide an adequate measurement foundation for subsequent structural model testing.

### Structural path analysis

**Figure 2:**
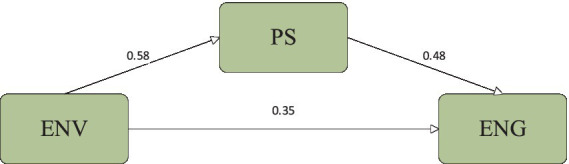
Standardized path coefficients in the SEM.

After the measurement model had been validated through confirmatory factor analysis, a structural equation model was constructed to examine the hypothesized relationships among perceived functional training environment, psychological safety, and training engagement. The model estimation results are presented in Figure 2.

The structural path results showed that perceived functional training environment was significantly and positively associated with psychological safety (*β* = 0.582, *p* < 0.001), supporting H1a. This finding suggests that athletes who perceive greater task clarity, appropriate challenge, and supportive feedback during training tend to report higher levels of psychological safety in the training context.

Psychological safety was also significantly and positively associated with training engagement (*β* = 0.476, *p* < 0.001), supporting H2a. This result indicates that athletes with higher psychological safety are more likely to demonstrate greater training effort, attentional focus, and identification with training tasks.

In addition, perceived functional training environment was significantly and positively associated with training engagement (*β* = 0.353, *p* < 0.001), supporting H3a. This finding suggests that athletes’ perceptions of the functional training environment are directly related to their training engagement and may also be indirectly associated with engagement through psychological safety.

To further examine the mediating role of psychological safety, the bootstrap method was used to test the indirect effect of perceived functional training environment on training engagement. A total of 5,000 bootstrap resamples were generated, and 95% confidence intervals were calculated. The standardized indirect effect of perceived functional training environment on training engagement through psychological safety was 0.277 (SE = 0.027, 95% CI [0.227, 0.330], *p* < 0.001). Because the confidence interval did not include zero, the mediating effect was statistically significant, supporting H4a.

The direct effect of perceived functional training environment on training engagement remained significant after psychological safety was included in the model (*β* = 0.353, SE = 0.039, 95% CI [0.275, 0.430], *p* < 0.001). This result indicates that psychological safety played a partial, rather than full, mediating role. The total effect was 0.630 (SE = 0.027, 95% CI [0.575, 0.680], *p* < 0.001). Based on the ratio of the indirect effect to the total effect, psychological safety accounted for 43.99% of the total effect (0.277/0.630), indicating that psychological safety represents an important psychological pathway linking perceived functional training environment to training engagement.

It should be noted that these findings were based on cross-sectional self-report data and a newly developed Functional Training Environment Perception Scale. Therefore, the results should be interpreted as evidence of statistical associations and indirect effects among variables, rather than as evidence of strict causal relationships.

### Moderating effects analysis

To further examine gender differences in the relationships among perceived functional training environment, psychological safety, and training engagement, multigroup structural equation modeling was conducted. Before comparing structural paths across gender groups, measurement invariance of the measurement model was tested. The results showed that both the configural invariance model and the metric invariance model met acceptable fit criteria, indicating that the male and female groups were comparable in terms of latent variable structure and primary measurement relationships. Further comparison showed that, relative to the configural invariance model, the absolute change in CFI for the metric invariance model did not exceed 0.01, and the change in RMSEA remained within an acceptable range. These results suggest that the measurement model was sufficiently stable across gender groups.

Based on this evidence, structural path coefficients were further compared between male and female athletes. It should be noted that the gender analysis in this study was conducted primarily to examine whether specific structural paths differed across groups. Differences in indirect effects between gender groups were not further tested. Therefore, the following interpretation is limited to gender differences in path strength within the present sample and should not be interpreted as evidence that gender moderates the overall mediating mechanism. The relevant results are presented in Table 3.

**Table 3:** Multi-group SEM results.

Grouping variable	Path	Female *β*	Male *β*	Δ*χ*^2^ (df = 1)	Significance
Gender	ENV → PS	0.66	0.51	4.21	*
Gender	PS → ENG	0.54	0.38	5.08	*
Gender	ENV → ENG	0.32	0.39	3.76	n.s.

*p < 0.05, **p < 0.01, ***p < 0.001; n.s., not significant.

As shown in Table 3, gender differences were mainly observed in the path from perceived functional training environment to psychological safety and in the path from psychological safety to training engagement. Specifically, the path coefficient from perceived functional training environment to psychological safety was higher among female athletes than among male athletes, and the between-group difference was statistically significant (*β*_female = 0.66, *β*_male = 0.51, Δ*χ*^2^ = 4.21, *p* < 0.05). The standardized between-group difference was 0.15. This result supports H1b, suggesting that gender may moderate the relationship between perceived functional training environment and psychological safety. In the present sample, female athletes showed a stronger association between their perceptions of the training environment and their psychological safety.

Similarly, the path coefficient from psychological safety to training engagement was higher among female athletes than among male athletes, and the between-group difference was statistically significant (*β*_female = 0.54, *β*_male = 0.38, Δ*χ*^2^ = 5.08, *p* < 0.05). The standardized between-group difference was 0.16. This result supports H2b, suggesting that gender may moderate the relationship between psychological safety and training engagement. In other words, the association between psychological safety and training engagement was stronger among female athletes in the present sample.

In contrast, the direct path from perceived functional training environment to training engagement did not differ significantly between gender groups (*β*_female = 0.32, *β*_male = 0.39, Δ*χ*^2^ = 3.76, *p* > 0.05). The standardized between-group difference was 0.07. This result does not support H3b, indicating that gender did not significantly moderate the direct relationship between perceived functional training environment and training engagement. Thus, the direct association between perceived training environment and training engagement appeared relatively consistent across male and female athletes.

Overall, the multigroup analysis indicates that gender differences were reflected primarily in the strength of specific structural paths rather than in the overall direction or basic structure of the model. Female athletes showed higher path coefficients for the pathways from perceived functional training environment to psychological safety and from psychological safety to training engagement. However, no significant gender difference was observed in the direct path from perceived functional training environment to training engagement. Because this study did not further test gender differences in bootstrap indirect effects, these findings should be interpreted as pathway-level group differences rather than as evidence that gender moderates the entire mediating process. Such differences may also be influenced by sport type, training experience, competitive level, and training evaluation context. Future studies should further examine these patterns using more refined grouping designs or models that include relevant control variables.

## Discussion

This study examined the relationships among perceived functional training environment, psychological safety, and training engagement. The findings showed that athletes’ positive perceptions of functional training task structure were significantly associated with higher psychological safety and greater training engagement. Psychological safety further mediated the relationship between perceived functional training environment and training engagement, while gender differences were mainly reflected in the strength of specific structural pathways.

Based on these findings, this study makes three theoretical contributions. First, by introducing psychological safety into functional training contexts characterized by high uncertainty, trial-and-error exposure, and frequent movement adjustment, this study extends the application of Psychological Safety Theory to competitive sport training research. Second, the study developed and preliminarily validated the Functional Training Environment Perception Scale, thereby providing a measurement basis for future research on athletes’ psychological experiences and behavioral responses in functional training contexts. Third, by using structural equation modeling, this study clarified the indirect pathway linking perceived functional training environment, psychological safety, and training engagement, while also examining the potential moderating role of gender in selected structural paths.

It should be emphasized that these contributions are based on cross-sectional self-report data and the present sample. Therefore, the findings should be interpreted as preliminary empirical evidence of statistical associations and indirect effects, rather than as definitive evidence of causal mechanisms.

### The uncertainty of functional training contexts: an explanation of the relationship between perceived task structure and psychological safety

The findings indicate a significant positive association between perceived functional training environment and athletes’ psychological safety. This relationship suggests that, in functional training contexts characterized by movement integration, adaptive task demands, and frequent trial-and-error exposure, psychological safety is closely related to how athletes interpret the training tasks themselves. When training goals are clearly defined, task challenges remain manageable, and feedback effectively supports movement correction, athletes may be more likely to regard trial and error, performance exposure, and temporary mistakes as normal components of the training process. In this sense, perceived functional training environment is associated with how training tasks are organized, experienced, and translated into relatively stable judgments of psychological safety.

The role of perceived functional training environment may be understood primarily through athletes’ interpretation of uncertainty. When athletes perceive the training context as goal-directed, appropriately challenging, and supported by constructive feedback, they may be better able to interpret task uncertainty as a manageable challenge rather than as an uncontrollable threat. This cognitive appraisal may reduce defensive responses during training and enable athletes to remain more open when attempting unfamiliar movements or adjusting existing techniques. As a result, athletes may develop a more stable sense of psychological safety. This interpretation is consistent with research suggesting that contextual support shapes psychological states by influencing individuals’ perceptions of task meaningfulness and controllability, rather than by directly determining behavior (Deci and Ryan, 2000; Ntoumanis, 2012).

These findings also extend discussions of the contextual antecedents of psychological safety. Previous research has primarily emphasized team interaction and leadership support as important sources of psychological safety (Edmondson, 1999; Edmondson and Lei, 2014). The present study suggests that, in competitive training contexts, the structure of the training task itself may also constitute a relevant contextual source. When training tasks are clear, challenging, and supported by corrective feedback, athletes may be less concerned about negative evaluation during trial-and-error learning and more likely to maintain psychological safety.

Therefore, the positive association between perceived functional training environment and psychological safety can be interpreted through the role of task structure in shaping athletes’ appraisal of uncertainty. When athletes perceive training tasks as understandable, challenges as manageable, and errors as correctable, uncertainty may be experienced as a learning-oriented space rather than merely as a source of pressure. This explanation is partly consistent with Self-Determination Theory, which emphasizes the importance of autonomy and competence in positive psychological experiences. However, because this study did not directly measure basic psychological need satisfaction, autonomy, competence, and relatedness should not be treated as empirically validated mediating mechanisms in the present model. A more cautious interpretation is that perceived functional training environment was statistically associated with psychological safety, and this association may be related to athletes’ subjective appraisals of task controllability, error correctability, and training supportiveness. In addition to perceived task structure, coaching feedback style, sport-specific training protocols, and athletes’ prior training experiences may also jointly shape their psychological safety.

### The mediating role of psychological safety: an explanatory pathway from situational perception to positive psychological experiences

The results indicate that psychological safety significantly mediates the relationship between perceived functional training environment and training engagement. This finding suggests that athletes’ psychological processing of the training context may be an important explanatory factor linking environmental perception to training engagement. In this process, psychological safety can be understood as a risk-buffering mechanism that regulates athletes’ responses to uncertainty, potential failure, and evaluative pressure. In highly uncertain and exploratory training contexts, behavioral engagement often involves the possibility of errors and external evaluation. When athletes remain highly sensitive to negative outcomes or interpret failure as evidence of insufficient ability, they may be more likely to avoid demanding tasks or reduce their level of participation. Conversely, when athletes perceive a high level of psychological safety, the perceived threat associated with potential mistakes may be reduced. They may therefore be more inclined to view trial and error as part of learning, which helps sustain their willingness to participate and remain engaged in training.

From a theoretical perspective, the mediating role of psychological safety may be explained through attentional resource allocation and emotional stability. On the one hand, when athletes are less concerned about negative evaluation or punishment during training, their attentional resources may be redirected from self-protection and evaluation concerns toward movement execution and task demands. This shift may help maintain concentration and behavioral consistency throughout the training process. On the other hand, higher psychological safety may support more stable emotional experiences when athletes face challenging tasks, thereby reducing the likelihood that temporary failure or performance errors will weaken their willingness to participate. These interpretations are broadly consistent with the emphasis on vigor, absorption, and positive psychological experiences in Work Engagement Theory (Schaufeli et al., 2002; Bakker and Demerouti, 2008). However, attentional resource allocation and emotional experience were not directly measured in this study. Therefore, these explanations should be regarded as theoretical interpretations rather than empirically verified process mechanisms.

In summary, the mediating role of psychological safety suggests that athletes’ safety appraisals of the training context may constitute an important psychological pathway linking perceived functional training environment to training engagement. Nevertheless, the present findings are based on cross-sectional self-report data and should therefore be interpreted as evidence of statistical associations and indirect effects rather than as evidence of causal mechanisms. Athletes’ sustained engagement in training may also be shaped by coaching style, training rhythm, organizational evaluation pressure, and individual motivational characteristics. Future research could incorporate process variables such as attentional resource allocation, emotional experience, and exploratory behavior to examine more directly how psychological safety may influence training engagement.

### The relationship between perceived safety and training engagement: a possible explanation based on positive psychological experiences

The findings further indicate a significant positive association between psychological safety and training engagement. This suggests that athletes’ safety appraisals within the training context may be closely related to their vigor, attentional focus, and identification with training tasks.

The relationship between psychological safety and training engagement can be theoretically understood through reduced evaluation concerns and enhanced task focus. In functional training settings, athletes are required to repeatedly attempt new movement strategies and make corrections after errors or performance deviations. When athletes remain concerned that mistakes may lead to negative evaluation, their psychological resources may be redirected toward self-protection and risk avoidance, thereby weakening their engagement with the training task itself. Conversely, when athletes perceive the training environment as psychologically safe, they may be less distracted by evaluative concerns and better able to concentrate on movement execution, technical adjustment, and task completion. In this sense, psychological safety may provide a stable psychological basis for sustained training engagement.

This association may also be explained through emotional experience and exploratory behavior. When athletes do not interpret mistakes solely as evidence of inadequate ability or as threats of evaluation, they may be better able to maintain emotional stability when facing training challenges. They may also be more willing to continue experimenting with and adjusting movement strategies. In functional training, active experimentation, continuous refinement, and movement exploration are central expressions of engagement. Psychological safety may therefore support sustained participation by reducing the perceived psychological cost of attempting unfamiliar behaviors.

It should be emphasized that attentional resource allocation, emotional experience, and exploratory behavior were not directly measured in the present model. Therefore, this study cannot demonstrate that psychological safety influences training engagement through these specific processes. Rather, it provides evidence of a significant positive association between psychological safety and training engagement. The explanations above should be understood as theoretical interpretations derived from Psychological Safety Theory and Work Engagement Theory. Future research should further test these potential mechanisms using longitudinal designs, experimental approaches, or multivariate mediation models.

### Contextual differences and relationship boundaries: a cautious interpretation of gender differences

Based on the preceding analysis, the results indicate that gender differences were observed in selected path coefficients among perceived functional training environment, psychological safety, and training engagement. Specifically, in the present sample, the path from perceived functional training environment to psychological safety and the path from psychological safety to training engagement were stronger among female athletes. By contrast, the direct path from perceived functional training environment to training engagement did not differ significantly between male and female athletes. These findings suggest that gender differences were reflected mainly in the strength of specific associations rather than in fundamental changes in the overall mechanism. However, because the invariance tests in this study were primarily conducted at the configural and metric levels, and because between-group path differences may still be influenced by sport type, training experience, and evaluation context, these findings should be interpreted cautiously.

From a mechanistic perspective, these differences may be related to athletes’ sensitivity to risk and evaluative information in uncertain task contexts. Functional training creates high-uncertainty and exploratory conditions in which athletes must repeatedly attempt, adjust, and correct movements. This process is often accompanied by potential failure and external evaluation. Existing research suggests that individual differences in sensitivity to social evaluation may shape risk appraisal and behavioral responses. When athletes are more sensitive to evaluative cues, they may rely more strongly on contextual support, thereby strengthening the association between perceived training environment and psychological safety.

The stronger association between psychological safety and training engagement among female athletes suggests that, in this sample, female athletes may depend more on contextual safety when translating psychological experiences into behavioral participation. When athletes experience higher psychological safety during training, they may be more likely to maintain positive emotional states and stable attentional engagement, which may support stronger behavioral participation. This interpretation is consistent with research emphasizing the role of psychological safety in promoting individual participation (Edmondson and Lei, 2014) and with sport-context studies showing that supportive environments are associated with athletes’ engagement (Fransen et al., 2020).

Notably, the direct association between perceived functional training environment and training engagement did not differ significantly by gender. This finding suggests that the direct link between environmental perception and behavioral engagement may have a degree of cross-gender consistency, whereas gender differences may be more evident in the psychological processes through which environmental perceptions are translated into engagement. In other words, athletes of different genders may not differ substantially in their direct behavioral responses to similar training environments; rather, variation may emerge in how they psychologically process those environments. These findings highlight the importance of considering both contextual characteristics and individual differences when explaining training engagement. At the same time, the observed group differences may also be intertwined with differences in sport distribution, training experience, and evaluation contexts. They should therefore be understood as boundary-level empirical patterns rather than as effects attributable to gender alone.

In summary, gender appears to function as a boundary condition in the mechanism model proposed in this study. It may influence the extent to which perceived functional training environment is associated with psychological safety and the extent to which psychological safety is associated with training engagement. This finding suggests that, in high-uncertainty training contexts, individual differences may not alter the basic direction of the mechanism but may shape its strength. Therefore, interpretations of training behavior should integrate both mechanism generality and individual variability.

## Conclusions and recommendations

### Conclusion

Based on questionnaire data from 468 active athletes, this study constructed and tested a theoretical model linking perceived functional training environment, psychological safety, and training engagement. The main findings are as follows.

First, perceived functional training environment was significantly and positively associated with both psychological safety and training engagement. Athletes who reported clearer training tasks, more appropriate task challenges, and more supportive feedback also tended to report higher levels of psychological safety and training engagement.

Second, psychological safety significantly mediated the relationship between perceived functional training environment and training engagement. This finding suggests that, in functional training contexts characterized by high uncertainty and frequent trial-and-error exposure, athletes’ positive perceptions of the training environment may be associated with stronger training engagement through enhanced psychological safety.

Third, multigroup analysis showed that gender differences were reflected mainly in the strength of specific structural paths rather than in fundamental changes in the direction or overall structure of the model. Specifically, female athletes showed higher path coefficients for the paths from perceived functional training environment to psychological safety and from psychological safety to training engagement. By contrast, the direct path from perceived functional training environment to training engagement did not differ significantly between male and female athletes. Because this study did not further test gender differences in indirect effects, these findings should be interpreted as group differences at the specific-path level rather than as evidence that gender moderates the entire mediating mechanism.

### Recommendations

Based on the mechanisms identified in this study, enhancing training engagement should not be understood simply as increasing training load or the number of training tasks. Rather, it requires optimizing training contexts and psychological experiences so that athletes can develop a stable sense of psychological safety. Specifically, practical improvements may be made in the following areas.

#### Clarify training task structure to reduce the cognitive burden of uncertainty

The findings indicate that perceived functional training environment is significantly associated with psychological safety, suggesting that task clarity and appropriate difficulty may influence athletes’ risk appraisals of the training context. Therefore, coaches should provide clear movement objectives and execution standards while maintaining an appropriate level of challenge. For example, in multi-joint or complex movement training, key movement components can be broken down, evaluation criteria can be clarified, and staged completion standards can be established. These strategies may provide athletes with a more stable basis for judgment when facing complex tasks and reduce anxiety arising from uncertainty and potential failure.

#### Optimize risk interpretation through process-oriented feedback

The development of psychological safety is closely related to how athletes interpret errors and feedback. Therefore, training feedback should avoid single-dimensional evaluations based solely on whether a movement is “right” or “wrong.” Instead, coaches should adopt process-oriented feedback that frames technical errors as necessary components of movement adjustment. For instance, feedback can emphasize “current-stage optimization directions” rather than “insufficient ability” and provide specific pathways for technical improvement. This approach may help athletes interpret training uncertainty as an opportunity for improvement, thereby reducing defensive responses during practice.

#### Establish low-risk trial spaces to encourage exploratory behavior

Functional training depends heavily on trial and error, adjustment, and movement reorganization. However, when training environments remain highly evaluative and exposing, athletes may narrow their range of attempts because of concerns about failure. Therefore, training sessions should include practice segments with reduced evaluation pressure. For example, before formal assessments or high-intensity training tasks, coaches may allocate time for free adjustment, allowing athletes to test different movement strategies without linking their performance to scores, rankings, or formal evaluation. Such structured arrangements may reduce the psychological cost of attempting unfamiliar behaviors and encourage greater movement exploration, thereby supporting training engagement.

#### Maintain psychological energy through appropriate pacing and task progression

Training engagement depends not only on motivation but also on athletes’ psychological resource states during training. In continuous high-intensity or highly complex training sessions, task difficulty should be increased progressively, and training pace should be adjusted to prevent prolonged exposure to high-pressure demands. For example, highly challenging tasks can be alternated with more stable or familiar exercises. This arrangement may help athletes maintain a sense of control at different stages of training while sustaining attention, effort, and energy investment.

#### Adapt support strategies to individual characteristics

The findings suggest that gender differences may exist in specific psychological pathways. In practice, coaches may adjust feedback intensity and interaction style according to athletes’ sensitivity to evaluation and risk. For athletes who are more sensitive to evaluation, coaches can increase process-based affirmation and specific guidance while reducing vague or emotionally charged judgments. For athletes with a stronger sense of autonomy, coaches may provide more challenging tasks and greater opportunities for self-directed adjustment. Differentiated support may help athletes with different characteristics develop a more stable foundation of psychological safety.

## Limitations

Although this study preliminarily identified psychological pathways linking perceived functional training environment, psychological safety, and training engagement in high-uncertainty training contexts, several limitations should be acknowledged.

First, this study adopted a cross-sectional questionnaire design. Athletes reported their perceptions of the functional training environment, psychological safety, and training engagement at a single time point. Therefore, the findings primarily reflect statistical associations among variables and do not permit strict causal inference. Although anonymous administration, item separation, and statistical tests for common method bias were used to reduce potential methodological bias, future research should further examine the stability and directionality of the proposed model through longitudinal designs, training interventions, or multi-source data.

Second, the Functional Training Environment Perception Scale was developed specifically for the context of this study. Although the scale demonstrated acceptable reliability and validity in the current sample, its applicability should be further validated across different sports, competitive levels, and training systems. In addition, this study focused mainly on testing the core theoretical pathways and did not include background variables such as sport type, training experience, or competitive level in the main model. Because the anonymous survey did not fully record school, team, or training-group identifiers, potential team-level dependencies could not be examined. The gender analysis was also based primarily on comparisons of structural path coefficients, without further testing gender differences in indirect effects. Accordingly, the relevant conclusions should be limited to the path associations and partial differences in path strength observed in the present sample. Future studies may employ control-variable models, more refined multigroup analyses, or hierarchical modeling to further test these relationships.

## Data Availability

The original contributions presented in the study are included in the article/Supplementary material, further inquiries can be directed to the corresponding author.

## References

[ref1] Appleton P. R. Ntoumanis N. Quested E. Viladrich C. Duda J. L. (2016). Initial validation of the coach-created empowering and disempowering motivational climate questionnaire (EDMCQ-C). Psychol. Sport Exerc. 22, 53–65. doi: 10.1016/j.psychsport.2015.05.008

[ref2] Bakker A. B. Demerouti E. (2008). Towards a model of work engagement. Career Dev. Int. 13, 209–223. doi: 10.1108/13620430810870476

[ref3] Bompa T. O. Buzzichelli C. A. (2019). Periodization: Theory and Methodology of Training. 6th Edn. Champaign, IL: Human Kinetics.

[ref4] Boyle M. (2016). New Functional Training for Sports. 2nd Edn. Champaign, IL: Human Kinetics.

[ref5] Cheung G. W. Rensvold R. B. (2002). Evaluating goodness-of-fit indexes for testing measurement invariance. Struct. Equ. Model. 9, 233–255. doi: 10.1207/S15328007SEM0902_5

[ref6] Cook G. (2010). Movement: Functional Movement Systems: Screening, Assessment, Corrective Strategies. Aptos, CA: On Target Publications.

[ref7] Deci E. L. Ryan R. M. (2000). The “what” and “why” of goal pursuits: human needs and the self-determination of behavior. Psychol. Inq. 11, 227–268. doi: 10.1207/S15327965PLI1104_01

[ref8] Edmondson A. (1999). Psychological safety and learning behavior in work teams. Adm. Sci. Q. 44, 350–383. doi: 10.2307/2666999

[ref9] Edmondson A. C. Lei Z. (2014). Psychological safety: the history, renaissance, and future of an interpersonal construct. Annu. Rev. Organ. Psychol. Organ. Behav. 1, 23–43. doi: 10.1146/annurev-orgpsych-031413-091305

[ref10] Fransen K. McEwan D. Sarkar M. (2020). The impact of identity leadership on team functioning and well-being in team sport: is psychological safety the missing link? Psychol. Sport Exerc. 51:Article 101763. doi: 10.1016/j.psychsport.2020.101763

[ref9001] Guo Z. Yang J. Wu M. Xu Y. Chen S. Li S. (2022). The associations among athlete gratitude, athlete engagement, athlete burnout: A cross-lagged study in China. Frontiers in Psychology, 13, 996144. doi: 10.3389/fpsyg.2022.996144 36248495 PMC9557925

[ref11] Hu L. T. Bentler P. M. (1999). Cutoff criteria for fit indexes in covariance structure analysis: conventional criteria versus new alternatives. Struct. Equ. Model. 6, 1–55. doi: 10.1080/10705519909540118

[ref12] Isoard-Gautheur S. Guillet-Descas E. Lemyre P. N. (2012). A prospective study of the influence of perceived coaching style on burnout propensity in high-level young athletes. J. Appl. Sport Psychol. 24, 20–34. doi: 10.1080/10413200.2011.574653

[ref13] Kahn W. A. (1990). Psychological conditions of personal engagement and disengagement at work. Acad. Manag. J. 33, 692–724. doi: 10.2307/256287

[ref14] Kline R. B. (2016). Principles and Practice of Structural Equation Modeling. 4th Edn. New York, NY: Guilford Press.

[ref15] Liu J.-D. Wu J.-X. Zou Y.-D. Wang Z.-H. Zhang S. Hu J.-C. . (2024). Development and initial validation of the engagement in athletic training scale. Front. Psychol. 15:1402065. doi: 10.3389/fpsyg.2024.1402065, 39108426 PMC11301410

[ref16] Lonsdale C. Hodge K. Jackson S. A. (2007). Athlete engagement: II. Development and initial validation of the athlete engagement questionnaire. Int. J. Sport Psychol. 38, 471–492. doi: 10.1037/t50268-000

[ref9002] Luo Y. Li S. Cao Y. Luo Z. (2025). The predictive role of coach–athlete relationship quality in training engagement and skill development among adolescent basketball players. Frontiers in Psychology 16, 1648082. doi: 10.3389/fpsyg.2025.1648082 40949347 PMC12426250

[ref17] Ntoumanis N. (2012). “A self-determination theory perspective on motivation in sport and physical education: current trends and possible future research directions,” in Advances in Motivation in Sport and Exercise, eds. Roberts G. C. Treasure D. C.. 3rd ed (Champaign, IL: Human Kinetics), 91–128.

[ref18] Ryan R. M. Deci E. L. (2017). Self-Determination Theory: Basic Psychological Needs in Motivation, Development, and Wellness. New York, NY: Guilford Press.

[ref19] Schaufeli W. B. Salanova M. González-Romá V. Bakker A. B. (2002). The measurement of engagement and burnout: a two sample confirmatory factor analytic approach. J. Happiness Stud. 3, 71–92. doi: 10.1023/A:1015630930326

[ref20] Taylor J. Collins D. Ashford M. (2022). Psychological safety in high-performance sport: contextually applicable? Front. Sports Act. Living 4:823488. doi: 10.3389/fspor.2022.823488, 35615347 PMC9125081

[ref9003] Van Yperen N. W. (2025). Athletes’ basic psychological need satisfaction and autonomous motivation: Differences between individual vs. team sports. Frontiers in Sports and Active Living 7, 1592356. doi: 10.3389/fspor.2025.1592356 41306187 PMC12643986

